# GetGenome: Overcoming inequalities in access to genomics technology

**DOI:** 10.1371/journal.pbio.3002804

**Published:** 2024-08-26

**Authors:** James Canham, Joe Win, GetGenome Network, Sophien Kamoun

**Affiliations:** 1 The Sainsbury Laboratory, Norwich Research Park, Norwich, United Kingdom; 2 GetGenome, Norwich Research Park, Norwich, United Kingdom

## Abstract

Although genomics has become integral to life science research, inequitable access to genomics technology remains prevalent. GetGenome, a non-profit organization, aims to overcome this by providing equitable access to genomics technology and training.

## Abstract 2

Although genomics has become integral to life science research, access to genomics technology remains inequitable. This article introduces GetGenome, a non-profit organization that aims to overcome this by providing global and affordable access to genomics technology and training.

The genomics era has been driven largely by technological advances that have resulted in a dramatic reduction in the cost of DNA sequencing [1]. However, many research communities still find DNA sequencing prohibitively expensive, time-consuming and technically challenging, leading to inequitable access to genomics technology and reduced scientific and commercial output. GetGenome is a newly formed non-profit organization (registered charity in England and Wales) that provides equitable access to genomics technology and genomics-related training, enabling researchers without access to genome sequencing to transition into the post-genomics stages of their projects without incurring costs.

## It started with just one student

During the COVID-19 pandemic in 2020, one of us (SK) was invited to attend the online thesis defense (viva) of Manel Chaouachi, a Tunisian PhD student. One aspect of the student’s project involved polymerase chain reaction (PCR) amplification of regions of interest that were hypothesized to occur in the genomes of plant-associated bacteria, with the results often inconclusive. However, when SK offered to sequence the genomes of the bacterial strains, it became evident that these regions that were challenging to amplify were simply absent in the studied strains. At the cost of only a few dozen pounds, the acquisition of genome sequences saved months of unfruitful work and helped to generate new hypotheses.

## GetGenome: Genomics and open science for all

This experience, and others like it, led us to formulate GetGenome as a bottom-up concept enabling biologists across world to access genomics technology. GetGenome provides genome sequences, including assembled genomes at no cost to the researcher and assists with the analysis and publication of the genome data (Box 1).

**Box 1. GetGenome: How it works**

- DEPOSIT your organisms for sequencing in GetGenome’s sample depository.
- ACCESS and learn to analyze the genomics data of your organisms.
- SHARE and publish the data using open science principles.
- NETWORK with other biologists to make the most out of the data.

Collaborations are often sought to surmount barriers to access genomics technology, although this can lead to compromises in autonomy and data ownership. In some cases, scientists from the Global South who contribute samples to their western colleagues may not even be recognized by authorship [2]. In-house genomics platforms can be developed to bolster local capacity, but they can incur exorbitant upkeep costs, unreliable access to reagents, and dependence on trained personnel, limiting their usefulness and sustainability. These challenges can be resolved by outsourcing DNA sequencing needs. A reasonable question is whether shipping costs would outweigh the benefits of such a scheme, but for our initial calls for projects in Tunisia, Mexico, and Pakistan, the shipping costs have been immaterial, accounting for approximately 0.5% of the total cost of each call, partly due to consolidation of samples at centralized hubs.

GetGenome delivers training workshops in basic genomics analysis and data-handling to build the necessary skills to interpret the data. Scientists are trained on their own genomics data, making them highly motivated and eager to learn. We aim to streamline training activities and resources through online tutorials and a seminar series. One of the aims of the workshops is to help participants to identify future training needs and gaps in their understanding. After acquiring new skills and gaining access to genomics data via GetGenome, researchers from the Centre de Biotechnologie de Borj Cédria (CBBC), in collaboration with the Tunisia Association of Genetic Resources, independently organized several workshops to address the needs of the local community, effectively extending the reach of GetGenome-led training.

We believe that scientific research should be made available to the public without charge and that scientists should be able to publish their data for the benefit of everyone without incurring costs. Therefore, GetGenome training activities include making our partners aware of the benefits of open science publishing. GetGenome assists participants to deposit their data in open genomics databases, as well as to concurrently publish a genome announcement “mini-paper” in the free-to-publish and free-to-access repository, Zenodo. This allows researchers to produce a citable, permanent scholarly output associated with a digital object identifier (DOI), enhancing visibility, garnering attention, and establishing credibility in their field, without impeding future journal publication [3].

GetGenome has developed a Material Transfer Agreement that reflects our dedication to fostering productive and ethical collaborations [4]. We do not conduct research with, or co-author articles produced by those we support, nor do we utilize or benefit directly from the genetic resources, placing our operations out-of-scope for the Nagoya Protocol on Access to Genetic Resources [5].

## GetGenome to date

**Fig 1:**
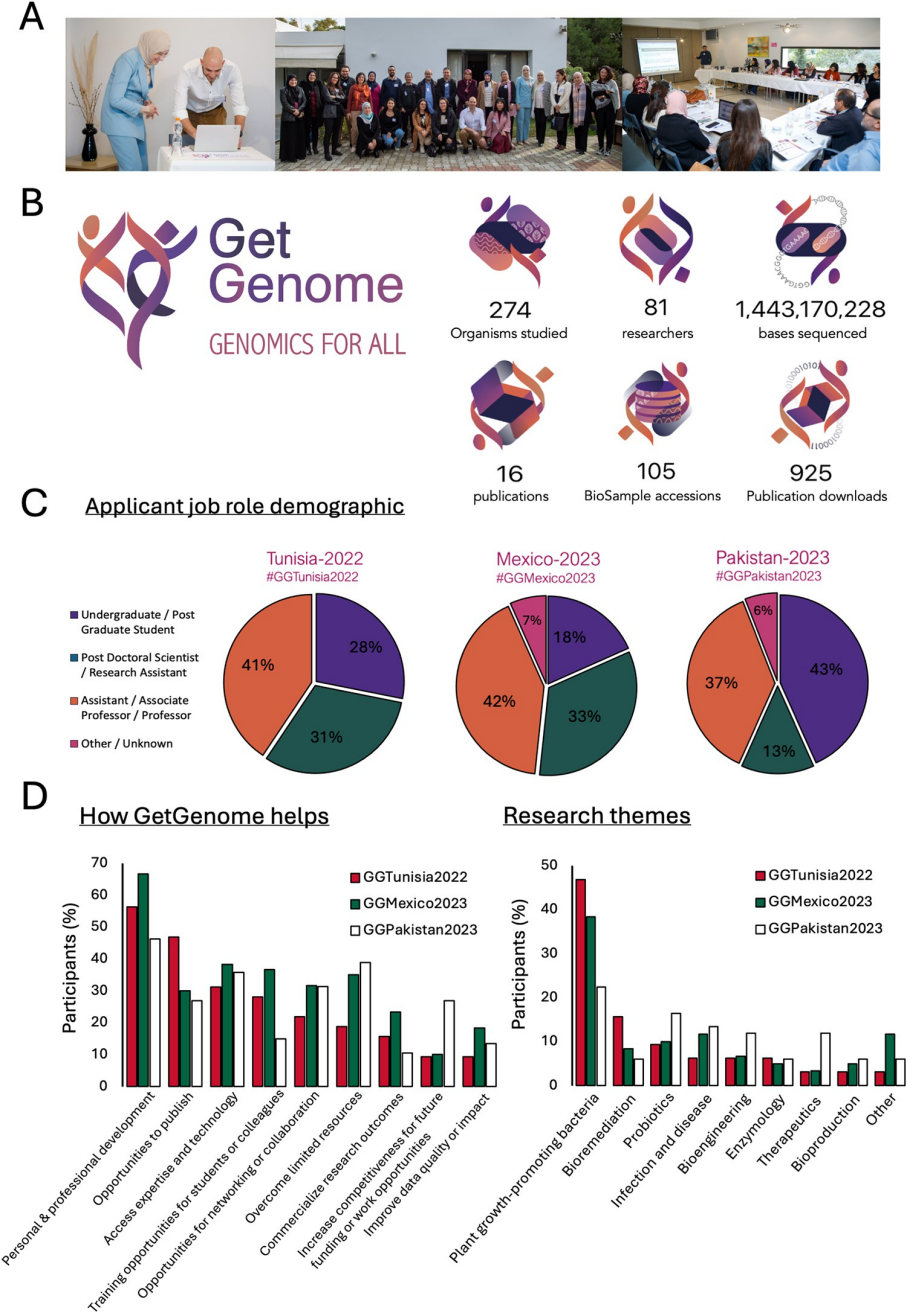
GetGenome’s achievements to date. **(A)** Images from the inaugural GetGenome Symposium held in Tunis, Tunisia, in December 2023. Photographs by James Canham. **(B)** The GetGenome logo and GetGenome outputs since its conception in 2022. Images created by Hsuan Pai [6]. **(C)** GetGenome calls for projects are designed to identify researchers facing barriers to accessing genomics technology and/or training. GetGenome’s calls for projects were launched in Tunisia, Mexico, and Pakistan during 2022–23 to support projects concerned with non-pathogenic bacteria. Charts show the job roles of applicants to GetGenome. **(D)** How GetGenome helps: Applicants were asked to state the how they might benefit from GetGenome support. These data are broadly summarized into nine of the most commonly mentioned benefits. Research theme of applicants to GetGenome’s calls for projects during 2022–23. Data are based on 159 applications to GetGenome’s calls for projects open during 2022–23.

To date, GetGenome has enabled the genome sequencing of over 250 organisms and dozens of plasmids, providing data to over 80 researchers in nine countries (Fig 1). GetGenome’s calls for projects were launched in Tunisia, Mexico, and Pakistan during 2022–23, focusing on non-pathogenic bacteria. The pool of applicants was diverse and over 50% of participants were early-career researchers (undergraduate or post-graduate students, post-doctoral researchers, or research assistants) (Fig 1).

Calls for projects are coordinated and managed locally by a committed team of GetGenome associates with an understanding of the needs of the research community, without participation from the UK-based team. The committee helps to disseminate the call, independently assess applications, and ensure the smooth importation of materials to GetGenome via local hubs.

In Tunisia, applicants were particularly intrigued by the opportunity to publish their findings, recognizing the significance of sharing their research with the wider scientific community. In Mexico, a spirit of knowledge-sharing prevailed, with applicants eager to use their newfound expertise to benefit their students and peers. Additionally, participants represented a broad geographic diversity, breaking away from the typical focus on Western-Mexican collaborations centered on Mexico City. In Pakistan, the prospect of overcoming resource limitations through GetGenome support proved to be a key motivating factor.

GetGenome also supports ground-breaking projects aimed at addressing pressing global challenges, such as the Wheat Disease Early Warning Advisory System (Wheat DEWAS) project launched by The International Maize and Wheat Improvement Centre (CIMMYT) and funded through a US$7.3 million grant from the Bill & Melinda Gates Foundation and the United Kingdom’s Foreign, Commonwealth & Development Office to enhance crop resilience to wheat diseases in Africa and South-East Asia [7].

GetGenome recently launched GGPlasmidSeq, which provides free plasmid sequencing for researchers based in over 100 low- and middle-income countries. This campaign is designed to foster research best-practice—e.g., by offering scientists the capacity to quality check and sequence their plasmids before engaging in long-term experiments. To date, we have helped to sequence dozens of plasmids, assisting researchers in Turkey, Thailand, Pakistan, and Argentina.

## The future for GetGenome

So far, our calls have been limited to non-pathogenic bacteria for logistical reasons and to enable us to test our operations with relatively cheap and easy-to-handle organisms. However, to address community needs, we will expand to a wider range of organisms, including fungi and various microbial pathogens. For similar reasons, we currently deploy Illumina sequencing, which is sufficient for most campaigns on non-pathogenic bacteria. However, we will use alternative technologies in the future when such technology is required, for example, when we tackle other organisms.

We will also consider open calls focused on specific taxa, research themes, or global challenges. Such campaigns will enable GetGenome to quickly pivot to support research communities whenever new needs or emergencies arise, similar to the Open Wheat Blast initiative [8,9].

GetGenome is still in its infancy and will continue to build its size and reach. Our more recent calls for projects such as in Latin America and the Middle East and North Africa in 2024 cover larger geographical regions and were created to rapidly expand the Network.

The GetGenome Network, providing researchers with unique opportunities for collaboration and knowledge exchange, will expand with the organization, enabling disparately located scientists working on similar organisms to network and collaborate. We have established an Alumni Network that will also provide a pipeline for trainees to become trainers to empower future cohorts. The Network will connect through newsletters, training and webinar sessions as well as symposia and conferences; opportunities that will grow and become more prevalent in the future.

As an independently registered spin-out from The Sainsbury Laboratory (TSL), we are optimistic about the sustainability of GetGenome, strengthened by TSL’s long-standing support from the Gatsby Charitable Foundation since the mid-1980s.

## Supporting information

S1 File Membership of GetGenome Network. (XLSX)
